# *In silico* system analysis of physiological traits determining grain yield and protein concentration for wheat as influenced by climate and crop management

**DOI:** 10.1093/jxb/erv049

**Published:** 2015-03-24

**Authors:** Pierre Martre, Jianqiang He, Jacques Le Gouis, Mikhail A. Semenov

**Affiliations:** ^1^INRA, UMR1095 Genetic, Diversity and Ecophysiology of Cereals, 5 chemin de Beaulieu, Clermont-Ferrand F-63100, France; ^2^Blaise Pascal University, UMR1095 Genetic, Diversity and Ecophysiology of Cereals, Aubière F-63177, France; ^3^Department of Computational and Systems Biology, Rothamsted Research, Harpenden, Herts AL5 2JQ, UK

**Keywords:** Crop growth model, genetic adaptation, grain protein concentration, grain yield, interannual variability, sensitivity analysis, yield stability, wheat (*Triticum aestivum* L.)

## Abstract

Genetic improvement of grain yield (GY) and grain protein concentration (GPC) is impeded by large genotype×environment×management interactions and by compensatory effects between traits. Here global uncertainty and sensitivity analyses of the process-based wheat model *SiriusQuality*2 were conducted with the aim of identifying candidate traits to increase GY and GPC. Three contrasted European sites were selected and simulations were performed using long-term weather data and two nitrogen (N) treatments in order to quantify the effect of parameter uncertainty on GY and GPC under variable environments. The overall influence of all 75 plant parameters of *SiriusQuality*2 was first analysed using the Morris method. Forty-one influential parameters were identified and their individual (first-order) and total effects on the model outputs were investigated using the extended Fourier amplitude sensitivity test. The overall effect of the parameters was dominated by their interactions with other parameters. Under high N supply, a few influential parameters with respect to GY were identified (e.g. radiation use efficiency, potential duration of grain filling, and phyllochron). However, under low N, >10 parameters showed similar effects on GY and GPC. All parameters had opposite effects on GY and GPC, but leaf and stem N storage capacity appeared as good candidate traits to change the intercept of the negative relationship between GY and GPC. This study provides a system analysis of traits determining GY and GPC under variable environments and delivers valuable information to prioritize model development and experimental work.

## Highlight

A global uncertainty and sensitivity analysis of the process-based model *SiriusQuality*2 was carried out to quantify the relationship between simple morpho-physiological traits and grain yield and protein concentration for wheat.

## Introduction

It has been estimated that in order to ensure food security, crop grain yields (GYs) should be increased globally by 70–100% within the next 40 years (Bruinsma, 2009). This means that the past relative rate of world GY increase, estimated at 0.5–1.7% for the major crops (Cassman, 2001; Tilman *et al.*, 2002; Ewert *et al.*, 2005), needs to be increased by ~40% (Tester and Langridge, 2010). However, in contrast to past GY increases, future GY improvements will have to be achieved in the context of global warming and with reduction of the use of water and fertilizers, because of environmental issues (Tilman, 1999; Godfray *et al.*, 2010). At the same time, grain protein concentration (GPC) will have to be maintained at its current level, since grain proteins are the major source of dietary proteins for humans, especially in developing countries, and since the economic value of most grain crops greatly depends on their GPC (Shewry, 2007). Maintaining GPC while increasing GY represents another challenge for plant breeders because of the commonly observed negative correlation between GY and GPC (e.g. Cooper *et al.*, 2001; Oury *et al.*, 2003; Aguirrezábal *et al.*, 2014).

Genetic improvements of GY and GPC are also impeded by their low heritability and by large genotype×environment×management interactions (Cooper *et al.*, 2001; Zheng *et al.*, 2009). Considerable efforts have been made to identify environmentally stable and genetically heritable traits and processes related to GY that can be used to guide crop breeding programmes, but only a few successful cases have been reported where identified traits or processes have led to genetic improvements of GY (Sinclair *et al.*, 2004; Yin and Struik, 2008; Passioura, 2010). The reasons lie in the many compensatory effects between traits (e.g. grain size versus grain number, light-saturated photosynthesis versus leaf surface area) and in the fact that complex traits such as GY and GPC are inherently determined at the canopy (community) level. Moreover improvements at the organ or plant level are often not translated at the canopy level (e.g. leaf level photosynthesis versus canopy radiation use efficiency; Long *et al.*, 2006). Therefore, quantitative analyses of the ‘trait hierarchy’ leading to GY and GPC improvements are needed (Sinclair *et al.*, 2004; Martre *et al.*, 2014a).

The use of virtual crops represented by a simulation model has been proposed to help molecular biologists, physiologists, and breeders to identify relevant traits and processes and to analyse their genetic determinism (e.g. Tardieu, 2003; Hammer *et al.*, 2006; Messina *et al.*, 2009; Bertin *et al.*, 2010). Crop simulation models can help develop hypotheses starting near the top of the ‘trait hierarchy’ leading to GY in the target environments (Sinclair *et al.*, 2004). Before addressing the question of how to translate the information of model simulations into knowledge that can be used by physiologists or geneticists, it is appropriate to have a better understanding of the model properties and behaviour. One of the best ways to do that is to conduct a global uncertainty and sensitivity analyses of the model to investigate the behaviour of the model in response to variations in model inputs (Cariboni *et al.*, 2007). By perturbing model parameters associated with simple physiological traits, uncertainty and sensitivity analyses allow investigation of crop responses and can help in identifying those traits that lead to a consistent high and stable GY or GPC in the target environments.

Most sensitivity analyses of crop simulation models have been performed in localized regions of the parameters’ space by varying one input parameter at a time, keeping the others at their nominal values, (Asseng *et al.*, 2002; Ruget *et al.*, 2002; Semenov *et al.*, 2009; Sinclair *et al.*, 2010). This simple approach does not take into account interactions between input parameters (i.e. their context dependency) and non-linear responses. Powerful numerical and statistical methods and tools for global model uncertainty and sensitivity analysis have been developed (Saltelli *et al.*, 2000), but until recently they have been overlooked by crop modellers and users. In the few studies where global sensitivity methods have been used to analyse crop simulation models, only a selected number of parameters, usually <20% of the total number, thought to be the most influential ones, were studied (Makowski *et al.*, 2006; Pathak *et al.*, 2007; Confalonieri, 2010; Confalonieri *et al.*, 2010a; Richter *et al.*, 2010). Moreover, with the exception of the study of Confalonieri *et al.* (2010b), the uncertainty associated with climate (site) and weather (year) variability was not considered. Consideration of all the input parameters of a model and their interactions as well as variations of environments (e.g. soil characteristics and climate) and crop management [e.g. nitrogen (N) fertilizer application] is required for a robust model evaluation.

Here, the results of a global uncertainty and sensitivity analysis of *SiriusQuality*2, a process-based model of wheat growth, conducted at three contrasted European sites using long- term weather data and two N treatments, are reported. The results show that GY and GPC are influenced by several traits and processes, and that the ranking of the traits depended on both the environment and N supply. The high level of parameter interactions indicated that the expression of the effect of a trait at the crop level also depends on the value of the other traits.

## Materials and methods

### The wheat simulation model *SiriusQuality*2

The wheat simulation model *SiriusQuality*2 (http://www1.clermont.inra.fr/siriusquality/) used in this study is a revised version of *SiriusQuality*1 (Martre *et al.*, 2006) and *Sirius* (Jamieson *et al.*, 1998b; Jamieson and Semenov, 2000). *SiriusQuality*2 has been calibrated and evaluated for several modern wheat cultivars and tested in many environments and climates, including conditions of climate change, and crop management scenarios (Martre et al., 2006, 2014b; Ferrise *et al.*, 2010; Asseng *et al.*, 2013, 2014).

*SiriusQuality*2 is a process-based model consisting of eight submodels that describe on a daily time step crop phenology (Phenology submodel), canopy development (Leaf Layer Expansion submodel), accumulation and partitioning of dry mass (DM; Light Interception and Use Efficiency and DM Allocation submodels), and N (N Allocation, and Root Growth and N Uptake submodels), including responses to shortage in the supply of soil water (Soil Drought submodel) and N (dealt with in the N Allocation submodel), and accumulation and partitioning of grain DM and N (Grain submodel). Two additional submodels describe crop evapotranspiration and soil N and water balances (Addiscott and Whitmore, 1991; Jamieson *et al.*, 1998b). The parameters of these two submodels were not investigated in this study, since they are related to soil properties and physical constants.

The Phenology submodel calculates the duration of six development phases, comprising pre-emergence (sowing to emergence), leaf production (emergence to flag leaf ligule appearance), flag leaf ligule appearance to anthesis, anthesis to the beginning of grain fill, effective grain filling, and maturation (Jamieson *et al.*, 1998a). The anthesis date is mainly determined by the rate of leaf production (1/phyllochron) and the final leaf number. The final leaf number is calculated by daylength (photoperiod) and temperature (vernalization) response subroutines (Brooking *et al.*, 1995; Jamieson *et al.*, 1998a; He *et al.*, 2012). Canopy development is simulated in the Leaf Layer Expansion submodel as a series of leaf layers associated with individual stems (Fournier *et al.*, 2005; Lawless *et al.*, 2005).

The Light Interception and Use Efficiency submodel calculates the amount of light intercepted by each leaf layer using the turbid medium approach (Monsi and Saeki, 2005) and uses it to produce biomass at an efficiency [radiation use efficiency (RUE)] calculated from temperature, air CO_2_ concentration, soil water deficit, leaf N mass per unit surface area (specific leaf N, SLN), and the ratio of diffuse to direct radiation (Jamieson *et al.*, 2000). The total above-ground biomass at any time is the sum of the daily rate of biomass accumulation of each leaf layer, which, in turn, is the product of RUE and intercepted photosynthetically active radiation by the leaf layers (Monteith, 1977). The canopy light extinction coefficient (*K*_L_) is assumed to be independent of N and water shortages (Robertson and Giunta, 1994). Part of the biomass produced each day is allocated to the stem so that a target constant specific DM of the leaf laminae (SLW_p_) and sheath (SSW_p_) is maintained. After the end of the endosperm cell division period, all new biomass plus a constant proportion of the labile leaf and stem biomass at the end of the endosperm cell division phase are allocated to grains.

Crop N uptake is driven by canopy expansion. The vertical distribution of leaf N follows the light distribution (Bertheloot *et al.*, 2008b), and the ratio of N to light extinction coefficients is determined by the crop N status (Moreau *et al.*, 2012). As for the biomass, any N not allocated to the leaves is allocated to the stem if its N concentration is less than its maximum. After anthesis, the capacity of the root system to take up N from the soil decreases linearly with thermal time (Martre *et al.*, 2006). After the end of the endosperm cell division stage, the rate of N transfer to grains is determined by the stem and leaf N concentrations, and follows first-order kinetics (Bertheloot et al., 2008a, b).

Seventy-five parameters are used to parameterize the processes described above (Table 1), which were subjected to the uncertainty and sensitivity analyses described below.

**Table 1.** Name, symbol, definition, nominal value, and unit of the 75 parameters of the wheat simulation model SiriusQuality2 All of the parameters belong to eight submodels of Phenology, Leaf layer expansion, Light interception and use efficiency, Grain, DM allocation, N allocation, Root growth and N uptake, and Soil drought factors.

Name	Symbol	Definition	Nominal value	Unit
Phenology				
Dse	*D*_se_	Thermal time from sowing to emergence	150	°Cd
MaxL	$L_{\text{max}}^{\text{abs}}$	Absolute maximum leaf number	18	Leaf
MinL	$L_{\text{min}}^{\text{abs}}$	Absolute minimum possible leaf number	8.7	Leaf
MaxLeafSoil	$L_{\text{max}}^{\text{soil}}$	Leaf number up to which the canopy temperature is equal to the soil temperature	4	Leaf
Lincr	$L_{\text{incr}}^{}$	Leaf number above which *P* is increased by Pincr	8	Leaf
Ldecr	$L_{decr}^{}$	Leaf number up to which *P* is decreased by Pdecr	2	Leaf
P	*P*	Phyllochron	100	°Cday
Pdecr	*P*_decr_	Factor decreasing the phyllochron for leaf number less than Ldecr	0.75	Dimensionless
Pincr	*P*_incr_	Factor increasing the phyllochron for leaf number higher than Lincr	1.25	Dimensionless
SLDL	SLDL	Daylength response of leaf production	0.15	Leaf h^–1^ (daylength)
PFLLAnth	$t_{\text{flag}}^{\text{anth}}$	Phyllochronic duration of the period between flag leaf ligule appearance and anthesis	3	Dimensionless
IntTvern	$T_{\text{int}}^{\text{ver}}$	Intermediate temperature for vernalization to occur	8	°C
MaxTvern	$T_{\text{max}}^{\text{ver}}$	Maximum temperature for vernalization to occur	17	°C
VAI	VAI	Response of vernalization rate to temperature	0.001	d^–1^ °C^–1^
VBEE	VBEE	Vernalization rate at 0°C	0.009	d^–1^
Leaf layer expansion				
AreaPL	$A_{{\text{L}}_{\text{N-1}}}^{\text{pot}}$	Maximum potential surface area of the penultimate leaf lamina	31	cm^2^ lamina^–1^
AreaSL	$A_{{\text{L}}_{S}}^{\text{pot}}$	Potential surface area of the leaves produced before floral initiation	2.56	cm^2^ lamina^–1^
AreaSS	$A_{{\text{S}}_{S}}^{\text{pot}}$	Potential surface area of the sheath of the leaves produced before floral initiation	1.83	cm^2^ sheath^–1^
PexpL	$t_{}^{\text{exp}}$	Phyllochronic duration of leaf lamina expansion	1.1	Dimensionless
PlagLL	$t_{{\text{L}}_{\text{N-1}}}^{\text{lag}}$	Potential phyllochronic duration between end of expansion and beginning of senescence for the leaves produced after floral initiation	6	Dimensionless
PlagSL	$t_{{\text{L}}_{S}}^{\text{lag}}$	Potential phyllochronic duration between end of expansion and beginning of senescence for the leaves produced before floral initiation	1.7	Dimensionless
PsenLL	$t_{{\text{L}}_{\text{N-1}}}^{\text{sen}}$	Potential phyllochronic duration of the senescence period for the leaves produced after floral initiation	9	Dimensionless
PsenSL	$t_{{\text{L}}_{S}}^{\text{sen}}$	Potential phyllochronic duration of the senescence period for the leaves produced before floral initiation	3.3	Dimensionless
RatioFLPL	$\alpha_{{\text{L}}_{N}{\text{/L}}_{\text{N-1}}}^{}$	Ratio of flag leaf to penultimate leaf lamina surface area	1	Dimensionless
aSheath	$\alpha_{\text{sheath}}$	Constant of the quadratic function relating the surface area of leaf sheath between two successive ligules and leaf rank after floral initiation	1.09	Dimensionless
NLL	η	Number of leaves produced after floral initiation	4.5	Leaf
Light interception and use efficiency				
Kl	*K*_L_	Light extinction coefficient	0.4	m^2^ (ground) m^–2^ (leaf)
FacCO2	$k_{{\text{CO}}_{\text{2}}}^{}$	Sensitivity of RUE to air CO_2_ concentration	0.3	Dimensionless
TauSLN	*k*_N_	Relative rate of increase of RUE with specific leaf N	1.9	m^2^ (leaf) g^–1^ (N)
SlopeFR	*k*_R_	Slope of the relationship between RUE and the ratio of diffuse to total solar radiation	1.5	Dimensionless
RUE	RUE	Potential radiation use efficiency under overcast conditions	3.4	g (DM) MJ^–1^
Tmax	$T_{\text{max}}^{\text{RUE}}$	Temperature at which RUE is null	50	°C
Topt	$T_{\text{opt}}^{\text{RUE}}$	Optimal temperature for RUE	18	°C
Grain				
Dcd	*D*_cd_	Duration of the endosperm cell division phase	250	°Cd
Der	*D*_er_	Duration of the endosperm endoreduplication phase	450	°Cd
Dgf	*D*_gf_	Grain filling duration (from anthesis to physiological maturity)	750	°Cd
Kcd	*k*_cd_	Relative rate of accumulation of grain structural DM	0.0084	(°Cd)^–1^
AlphaNC	α_N/C_	Grain structural N to C ratio	0.02	Dimensionless
EarGR	σ	Ratio of grain number to ear dry matter at anthesis	100	Grain g^–1^ (DM)
DM allocation				
Deg	*D*_eg_	Fraction of PFLLAnth for ear growth before anthesis (counted from flag leaf ligule appearance)	0.25	Dimensionless
SLWp	SLW_p_	Potential specific lamina DM	45	g (DM) m^–2^
SSWp	SSW_p_	Potential specific sheath DM	90	g (DM) m^–2^
FracLaminaBGR	γ_laminae_	Fraction of anthesis laminae DM allocated to the grain	0.25	Dimensionless
FracSheathBGR	γ_sheath_	Fraction of anthesis sheath DM allocated to the grain	0.25	Dimensionless
FracStemWSC	γ_wsc_	Fraction of anthesis stem DM in the water-soluble carbohydrate pool	0.1	Dimensionless
FracBEAR	μ	Fraction of biomass allocated to the ear during the ear growth period	0.5	Dimensionless
N allocation				
LLOSS	LLOSS	Fraction of leaf N resorption resulting in a reduction of LAI	0.6	m^2^ (leaf) m^–2^ (ground)
CritSLN	$N_{\text{cri}}^{\text{LA}}$	Critical area-based N content for leaf expansion	1.5	g (N) m^–2^ (leaf)
MaxSLN	$N_{\text{max}}^{\text{LA}}$	Maximum potential specific leaf N of the top leaf layer	2.2	g (N) m^–2^ (leaf)
MinSLN	$N_{\text{min}}^{\text{LA}}$	Specific leaf N at which RUE is null	0.35	g (N) m^–2^ (leaf)
StrucLeafN	$N_{\text{stru}}^{\text{LM}}$	Structural N concentration of the leaves	0.006	g (N) g ^–1^ (DM)
MaxStemN	$N_{\text{max}}^{\text{SM}}$	Maximum potential stem N concentration	0.0075	m (N) g^–1^ (DM)
StrucStemN	$N_{\text{stru}}^{\text{SM}}$	Structural N concentration of the true stem	0.005	g (N) g ^–1^ (DM)
AlphaKn	$\alpha_{K_{\text{N}}}^{}$	Scaling coefficient of the relationship between the ratio of N to light extinction coefficients and the N nutrition index	3.82	m^2^ (ground) m^–2^ (leaf)
AlphaSSN	$\alpha_{N_{\text{LA}}/N_{\text{ShA}}^{}}^{}$	Scaling coefficient of the allometric relationship between area-based lamina and sheath N mass	0.9	g (N) m^–2^
AlphaNNI	$\alpha_{\text{NNI}}^{}$	Scaling coefficient of the N dilution curve	5.35	10^2^ × g (N) g^–1^ (DM)
BetaKn	$\beta_{K_{\text{N}}}^{}$	Scaling exponent of the relationship between the ratio of N to light extinction coefficients and the N nutrition index	2.063	Dimensionless
BetaSSN	$\beta_{N_{\text{LA}}/N_{\text{ShA}}^{}}^{}$	Scaling exponent of the relationship between area-based lamina and sheath N mass	1.37	Dimensionless
BetaNNI	$\beta_{\text{NNI}}^{}$	Scaling exponent of the N dilution curve	0.442	Dimensionless
MaxLeafRRND	$\chi_{\text{leaf}}$	Maximum relative rate of leaf N depletion	0.004	(°Cd)^–1^
MaxStemRRND	$\chi_{\text{stem}}$	Maximum relative rate of stem N depletion	0.004	(°Cd)^–1^
Root growth and N uptake				
DMmaxNuptake	$C_{\max}^{\text{Nuptake}}$	Crop DM at which the potential rate of root N uptake equals MaxNuptake	100	g (DM) m^–2^
MaxRWU	*K*_max_	Maximum relative rate of root water uptake from the top soil layer	0.1	d^–1^
MaxNuptake	$N_{\text{pot}}^{\text{uptake}}$	Maximum potential rate of root N uptake	0.5	g (N) m^–2^ (ground) d^–1^
RVER	RVER	Rate of root vertical extension	0.001	m (°Cd)^–1^
BetaRWU	λ	Efficiency of the root system to extract water through the vertical soil profile	0.07	Dimensionless
Soil drought factors				
MaxDSF	DSF_max_	Maximum rate of acceleration of leaf senescence in response to soil water deficit	3.25	Dimensionless
LowerFTSWexp	$W_{\text{lower}}^{\text{exp}}$	Fraction of transpirable soil water for which the rate of leaf expansion equals zero	0.25	Dimensionless
LowerFTSWgs	$W_{\text{lower}}^{\text{gs}}$	Fraction of transpirable soil water for which the stomatal conductance equals zero	0.1	Dimensionless
LowerFTSWrue	$W_{\text{lower}}^{\text{RUE}}$	Fraction of transpirable soil water for which RUE equals zero	0	Dimensionless
LowerFTSWsen	$W_{\text{lower}}^{\text{sen}}$	Fraction of transpirable soil water value for which DSF_max_ is reached	0.1	Dimensionless
UpperFTSWexp	$W_{\text{upper}}^{\text{exp}}$	Fraction of transpirable soil water threshold for which the rate of leaf expansion starts to decrease	0.65	Dimensionless
UpperFTSWgs	$W_{\text{upper}}^{\text{gs}}$	Fraction of transpirable soil water threshold for which the stomatal conductance starts to decrease	0.5	Dimensionless
UpperFTSWrue	$W_{\text{upper}}^{\text{RUE}}$	Fraction of transpirable soil water threshold for which RUE starts to decrease	0.3	Dimensionless
UpperFTSWsen	$W_{\text{upper}}^{\text{sen}}$	Fraction of transpirable soil water threshold for which the rate of leaf senescence starts to accelerate	0.5	Dimensionless

### Simulation set-up

Two French sites, Avignon (AV) and Clermont-Ferrand (CF), and one UK site, Rothamsted (RR), were selected for this study (Table 2). The characteristics of the soil used at the three sites are summarized in Table 3. The soil water-holding characteristics at CF and RR were similar to that previously reported for these two sites (Jamieson *et al.*, 1998b; Martre *et al.*, 2006), while at AV parameters corresponding to a sandy soil were used to ensure water-limited conditions for most years. Simulations were conducted with historical weather data of 40 continuous years (1970–2009) at each site. Recommended sowing dates were used at each site (Table 2). The sowing density was 250 seeds m^–2^ at the three sites. Soil N characteristics (organic and inorganic N content) were the same at all sites (Table 3).

**Table 2.** Summary of the location and climate of the three sites used in this study Climatic data are median values for the 1970–2009 period at Avignon (AV), Clermont-Ferrand (CF), and Rothamsted (RR); values in square brackets are 0.75 and 0.25 quantiles. Crop emergence, anthesis, and physiological maturity dates were predicted using the wheat simulation model *SiriusQuality*2.

Site	Longitude (°)	Latitude (°)	Elevation (m a.s.l.)	Sowing date	Anthesis date	Emergence to anthesis			Anthesis to physiological maturity		
						Mean daily temperature (°C)	Cumulated solar radiation [MJ (DM) m^–2^]	Cumulated precipitation (mm)	Mean daily temperature (°C)	Cumulated solar radiation [MJ (DM) m^–2^]	Cumulated precipitation (mm)
AV	4.85	43.91	24	15 Nov.	11 May [6 May–15 May]	9.0 [8.5–9.4]	1744 [1647–1828]	255 [223–326]	19.1 [18.7–20.0]	1160 [1098–1213]	73 [45–109]
CF	3.10	45.80	329	01 Nov.	26 May [19 May–1 Jun]	7.1 [6.6–7.7]	1699 [1556–1778]	271 [175–294]	17.9 [17.2–18.6]	1080 [1003–1160]	91 [77–144]
RR	–0.35	51.80	128	10 Oct.	11 Jun. [5 Jun.–18 Jun.]	6.4 [6.1–6.8]	1622 [1500–1755]	446 [406–481]	15.4 [14.6–16.0]	1001 [948–1087]	110 [76–150]

**Table 3.** Summary of the soil characteristics at the three sites, Avignon (AV), Clermont-Ferrand (CF), and Rothamsted (RR), used in this study

Site	Maximum root depth (m)	N mineralization constant (×10^5^ d^–1^)	Top soil organic N [g (N) m^–2^]	Root zone inorganic N [g (N) m^–2^]	Percolation coefficient (dimensionless)	Soil textural class (USDA system)	Soil moisture characteristics [m^3^ (H_2_O) m^–3^ (soil)]			Available soil water content [mm (H_2_O)]
							Saturation	Field capacity	Permanent wilting point	
AV	1	2	1 000	4	0.7	Sandy	0.38	0.15	0.70	80
CF	1	2	1 000	4	0.4	Sandy loam	0.43	0.24	0.90	150
RR	1	2	1 000	4	0.3	Loam	0.47	0.33	0.14	190

Two N treatments were considered at each site, a high N treatment [HN, 20g (N) m^–2^] corresponding to the commercial N rate in the investigated environments with a late N application to optimize GPC, and a low N treatment [LN, 4g (N) m^–2^] corresponding to 20% of HN. For HN, N fertilizer was applied as four split dressings at the growth stages GS21 [4g (N) m^–2^], GS31 [8g (N) m^–2^], GS37 [6g (N) m^–2^], and GS65 [2g (N) m^–2^] described by the Zadoks scale (Zadoks *et al.*, 1974). For LN, N fertilizer was applied once at GS30. An irrigation of 10mm was applied at sowing to ensure good crop emergence conditions, and 5mm irrigations were applied on the dates of N applications to ensure dissolution of N fertilizer in the soil solution. This limited amount of water did not have any other effect on crop development or growth.

### Morris screening analysis

The 75 plant parameters of *SiriusQuality*2 were first analysed using the one-at-a-time (OAT) Morris method (Morris, 1991) to rank them in order of importance and to identify parameters involved in interactions with other parameters or parameters whose effect are non-linear.

The Morris method is based on individually randomized OAT experiments, in which the impact of varying each of the chosen parameters is evaluated in turn. In this method, each vector of parameters is first discretized into *p* levels corresponding to the quantiles of the parameter distribution and defining a *k*-dimensional and *p*-level region of experiment in the parameter space, where *k* is the number of parameters (Saltelli *et al.*, 2004). The sampling strategy proposed by Campolongo *et al.* (2007) was used, where *r* elementary effects are sampled to construct *r* trajectories of (*k*+1) points in the region of the experiment, each providing *k* elementary effects, one per input parameter. The number of model executions is thus equal to *r*×(*k*+1). Here, *p* was set to 8, and 760 vectors of parameters corresponding to 10 trajectories were generated using the uncertainty and sensitivity analysis software SimLab version 2.2 (Saltelli *et al.*, 2004). The parameters were assumed to be uniformly distributed in [0, 1] and then rescaled from the unit hypercube to their actual values using the following equation:

$$X_{i}^{m}=X_{i}^{d}\times (\Delta_{i}^{min}\;+\;(\Delta_{i}^{max}-\Delta_{i}^{min})\times \;P)$$ (1)

where $X_{i}^{m}$ and $X_{i}^{d}$ are the modified and default values of the parameter *x*_i_, respectively; $\Delta_{i}^{min}$ and $\Delta_{i}^{max}$ are the minimum and maximum fractional variations of the parameter *x*_i_, respectively; and *P* is a random number following a uniform distribution in [0, 1]. The space of each parameter was set to be ±20% of its default value, which encompass the observed range of genetic variation for most of the parameters (Martre *et al.*, 2007).

After running *SiriusQuality*2 for each of the parameter sets under each of the site–N treatment–year combinations, the model outputs were post-processed using SimLab. For each parameter, two sensitivity indices were obtained from the distribution of their elementary effects. The first is the mean of the distribution of the absolute values of the elementary effects (μ*), which estimates the overall importance of the parameter on the model outputs (Campolongo *et al.*, 2007). The second is the standard deviation of the distribution of elementary effects (σ), which indicates either a parameter interacting with other parameters or a parameter whose effect is non-linear.

In order to plot heat maps of μ* and σ, they were rescaled in [0, 1] since different output variables may have different magnitudes of μ* and σ. For each site–N treatment–output variable combination, the median of μ* and σ over the 40 years of simulation was first calculated and then rescaled using the formula:

$$z_{i,j}^{r}=\frac{z_{i,j}-z_{i,j}^{min}}{z_{i,j}^{max}-z_{i,j}^{min}}$$ (2)

where $z_{i,j}^{r}$, $z_{i,j}^{min}$, and $z_{i,j}^{max}$ are the rescaled, minimum and maximum values of μ* or σ for the *i*th parameter and *j*th output variable, respectively.

The sign of μ* was retrieved by conducting an independent OAT experiment. Each parameter was changed in turn by ±50% of its default value in 10% increments. Then for each parameter–site–N treatment combination, the slopes of the linear regression between the model outputs and the parameters were calculated and the sign of the slopes was given to the rescaled μ*. This provided useful information of the sign of the elementary effects, but it should be treated with caution since it might be significantly overestimated in the case of non-monotonic elementary effect distribution. For instance, a parameter with a large and negative μ* value should be considered as very significant, but the sign of the effect might be negative or positive depending on the position of the parameter input space where it is sampled, and its overall effect is therefore not necessarily negative. However, in a complex model such as *SiriusQuality*2, non-monotonic elementary effect distributions are infrequent (Saltelli *et al.*, 2008).

### Extended Fourier amplitude sensitivity test

The influential parameters identified by the Morris method were further analysed using the quantitative extended Fourier amplitude sensitivity test (E-FAST; Saltelli *et al.*, 1999). As for the Morris analysis, the space of each parameter was set to be ±20% of its default value assuming a uniform distribution. The *k* input parameters were coded so that their domain of variation was [0, 1]^k^. A total of 29 889 parameter vectors were generated using SimLab (i.e. 729 different positions in the input space were considered for each parameter), which is much higher than the minimum number of 2665 required by the E-FAST method with the number of parameters considered in this study. The generated random values of the parameters were then rescaled to their actual values using Equation 1, and *SiriusQuality*2 was run for all the site–N treatment–year combinations, resulting in 7.17×10^6^ model runs from which the Fourrier spectrum was calculated in SimLab on the different model outputs to estimate the first-order (*S*_i_) and total (*S*_Ti_) effect of each of the 41 influential parameters for each site–N treatment combination.

## Results

### Grain yield and protein concentration uncertainty and environment by N interactions

**Fig. 1.**
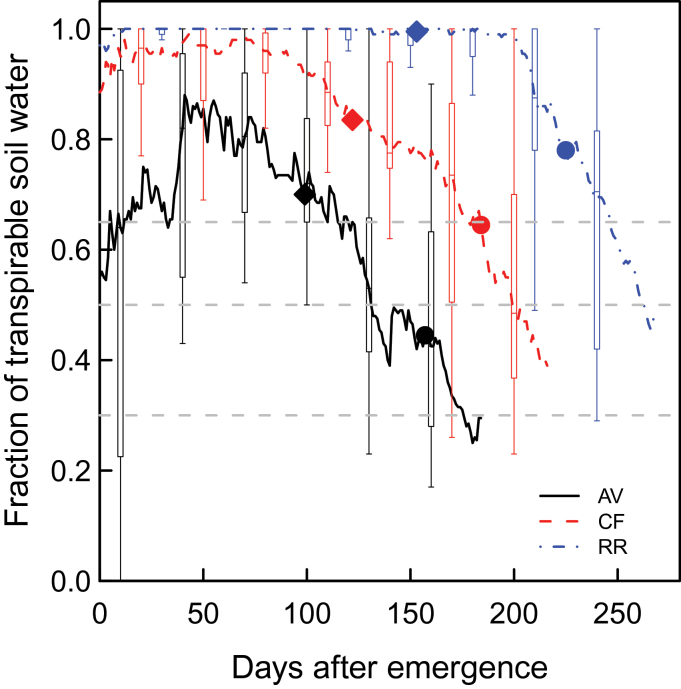
Fraction of transpirable soil water (FTSW) versus days after emergence at Avignon (AV; solid line), Clermont-Ferrand (CF; dashed line), and Rothamsted (RR; dash-dotted line) simulated with the wheat simulation model *SiriusQuality*2 under high N supply. Lines are median values for each site calculated for 40 years (1970–2009). Box plots show the interannual variability of FTSW calculated over the same period and plotted every 30 d (for the sake of clarity), the edges of the boxes represent the 25% and 75% percentiles, and the solid vertical bars the 10% and 90% percentiles. Filled diamonds and circles indicate the median of the beginning of stem extension (Zadoks stage GS31) and of the anthesis date (GS65), respectively. The horizontal dashed lines indicate the default values in *SiriusQuality*2 of the FTSW threshold for the responses of leaf expansion (UpperFTSWexp=0.65), leaf senescence and stomatal conductance (UpperFTSWgs and UpperFTSWsen=0.5), and biomass production (UpperFTSWrue=0.3). Simulations were performed using the default value of all the parameters (Table 1). (This figure is available in colour at *JXB* online.)

The three sites used for this study differed in terms of their climate (Table 2; Supplementary Fig. S1 available at *JXB* online) and soil (Table 3). AV has a Mediterranean climate with warm and dry summers and mild wet winters. CF has a Continental climate with hot summers and cold and relatively dry winters, while RR has an Oceanic climate with moderately cool summers and comparatively warm winters and evenly distributed precipitations. These sites are representative of the climate diversity of the wheat-growing regions in Europe. The site differences, as felt by the crops, are illustrated by the dynamics of the fraction of transpirable soil water (FTSW) during the growing season (Fig. 1). AV was characterized by high probabilities of mild water deficits early in the season and during the stem extension period, and of severe water deficits during grain filling. At this site, for 25% of the years, simulated FTSW decreased below 0.65, the threshold value for reduction of leaf expansion (LowerFSTWexpansion), after the beginning of stem extension, and for 55% of the years leaf senescence and stomatal conductance were significantly accelerated by water deficit (i.e. FTSW <0.5) during the grain-filling period. Comparably, almost no water limitation occurred during the vegetative growth period at RR. At this site, mild water deficits occurred during the second half of grain filling for 55% of the years, and for most of the years it had no significant effect on GY and GPC (data not shown). The situation at CF was intermediate to that at the other two sites. This site was characterized by late water deficit. Although at a given site the patterns of FTSW among the simulated years were similar (data not shown), the levels of water deficits were very variable (as illustrated by the box plots in Fig. 1). For example, at AV, the 10% and 90% percentiles of FTSW at anthesis were 0.31 and 0.87, respectively.

**Fig. 2.**
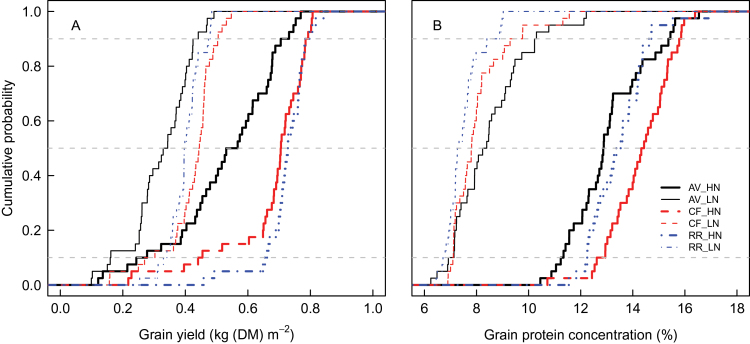
Cumulative probability distributions of simulated grain yield (A) and grain protein concentration (B) at Avignon (AV; solid lines), Clermont-Ferrand (CF; dashed lines), and Rothamsted (RR; dash-dotted lines) under high (HN; thick lines) and low (LN; thin lines) N supply simulated by the wheat simulation model *SiriusQuality*2 for 40 years (1970–2009). The *y*-axis refers to the probability that simulated yield (A) or grain protein concentration (B) is lower than a certain threshold yield or grain protein concentration, respectively (indicated by the *x*-axis). Simulations were performed using the default value of all the parameters (Table 1). The horizontal dashed lines are 10, 50 (median), and 90% percentiles. (This figure is available in colour at *JXB* online.)

Simulated GY was significantly lower and more variable at AV than at the two other sites (Fig. 2A), especially under HN, where it ranged from 0.14kg DM m^–2^ to 0.77kg DM m^–2^. Under HN, for most of the years, GY was similar at RR and CF, but under LN on average it was 6% higher at CF than at RR. At the three sites, GY was more variable under HN than under LN. On average GY was 88% higher under HN than under LN. As for GY, GPC was more variable at AV (ranging from 6.2% to 12.2% under LN and from 10.5% to 16.5% under HN) than at the two other sites. As for GY, the model predicted significant site×N interactions for GPC (Fig. 2B). Although it is usually observed that GPC is higher under elevated temperature or water deficit because carbon assimilation is more affected by these environmental constraints than N assimilation (Triboi and Triboi-Blondel, 2002; Aguirrezábal *et al.*, 2014), simulated GPC under HN was lower at AV than at the two other sites despite AV being a more stressful environment. This result can be explained by significant water deficit during the stem extension period at AV, which reduced the expansion of the canopy, and therefore limited N storage in vegetative tissues during the pre-anthesis period. Severe water deficits at this site also reduced soil N supply to the plant due to their negative effects on organic N mineralization. Under LN, GPC was higher at AV than at the two other sites, because of the strong limitation of crop growth and N accumulation, independently of the climate. For both N treatments, CF had higher GPC than RR. This was expected since CF had a higher probability of water deficit and supraoptimal temperature during grain filling.

### Screening of the model input parameters

The influence of all 75 parameters of the *SiriusQuality*2 model (Table 1) was first assessed using the Morris screening method (Morris, 1991). The perturbation of the parameters resulted in –14 d to +17 d differences in anthesis date (Supplementary Table S1 at *JXB* online). GY (–45% to +16%) and GPC (–20% to +35%) also showed large variations in response to parameter variations. The responses of eight key model outputs were analysed in detail. Grain protein deviation (GPD), namely the residual from the regression of GPC on GY, was also calculated (Monaghan *et al.*, 2001). Several authors have used GPD to identify genotypes with higher GPC than would be expected from their GY (e.g. Monaghan *et al.*, 2001; Oury and Godin, 2007; Bogard *et al.*, 2010). For each site–N–year combination, GPD was calculated by regressing GPC against GY for the 760 parameter combinations (‘genotypes’) generated in the Morris analysis. Under HN at AV the regression was significant in only 75% of the years and the median value of *r* ^2^ was only 0.05 (Supplementary Table S2). For the other site–N combinations, the correlations were highly significant in >95% of the years, and the ranges and distributions of the slope [median value –15.6% kg^–1^ (DM) m^2^] and *r* ^2^ (median value 0.53) were in close agreement with that calculated from experimental data (e.g. Oury *et al.*, 2003; Bogard *et al.*, 2010).

**Fig. 3.**
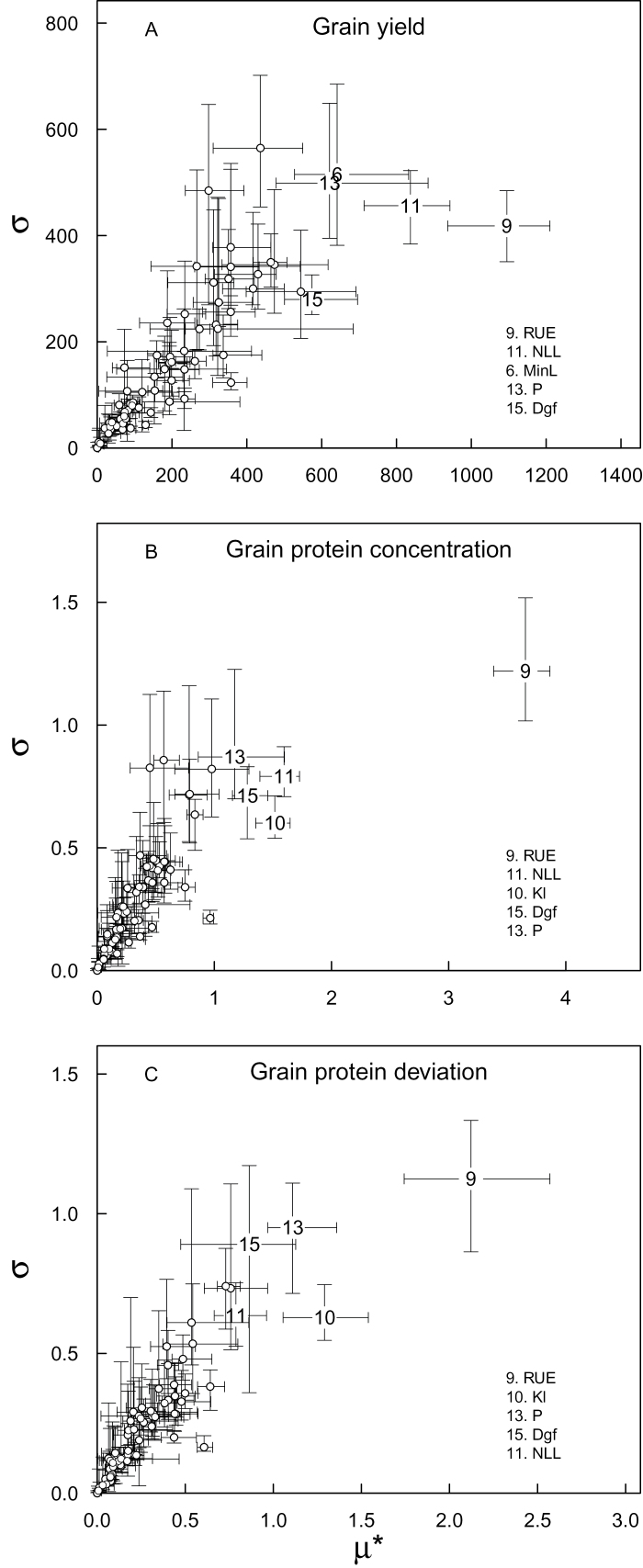
Median of the standard deviation (σ) versus median of the absolute mean (μ*) of the elementary effects for the 75 input parameters of the wheat simulation model *SiriusQuality*2 with respect to grain yield (A), grain protein concentration (B), and grain protein deviation (C) at Clermont-Ferrand under low N supply. The error bars are the 25% and 75% percentiles of σ and μ* for *n*=40 years, respectively. Only the five parameters with the highest μ* values are identified.

The ‘overall’ effect (μ*) and the non-linear and/or interaction effects (σ) of the parameters were well correlated for all model outputs, as illustrated in Fig. 3 for GY, GPC, and GPD at CF under LN. The influence of the parameters on the model outputs showed a high level of uncertainty associated with the year, and at a given site the ranking of the parameters depended strongly on the years.

**Fig. 4.**
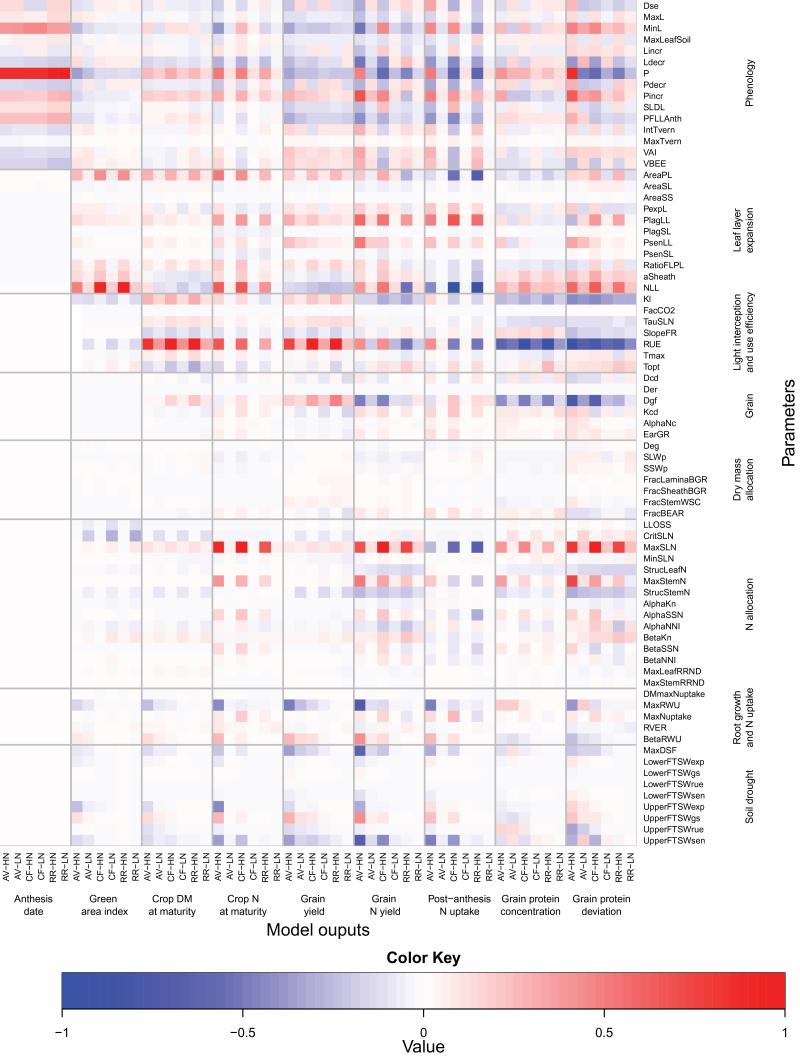
Heat map of the median values of the mean of the distribution of the absolute elementary effect (μ*) from the Morris screening analysis of the 75 input parameters of the wheat simulation model *SiriusQuality*2 on anthesis date, green area index, crop DM at maturity, crop N at maturity, grain DM at maturity, grain N at maturity, post-anthesis N uptake, grain protein concentration, and grain protein deviation. Simulations were performed at Avignon (AV), Clermont-Ferrand (CF), and Rothamsted (RR) under high (HN) and low N (LN) supplies for 40 years (1970–2009). The median of μ* was rescaled to [0, 1] across the sites and N treatments so for a given output they can be compared across the sites and N treatments. Negative values (blue colour) indicate that the parameter negatively influences the corresponding model output, and vice versa. The parameters were grouped according to the submodel to which they belong.

The median values of the rescaled (see the Materials and methods) ‘overall’ effect (Fig. 4) and of the non-linear and/or additive effects (Supplementary Fig. S2 at *JXB* online) are summarized on heat maps, which give a grand-view of parameter×site×N interactions. Anthesis date was most influenced, in order of importance, by the phyllochron (P) and the absolute minimum possible leaf number (MinL), the duration of the period between the appearance of the flag leaf ligule and anthesis (PFLLAnth), and the response of vernalization rate to temperature (VBEE; Fig. 4). Except for the maximum temperature for vernalization (MaxTvern), which had no significant overall effect on anthesis date, all the phenology parameters had significant overall and non-linear and/or interaction effects on canopy expansion and biomass and N accumulation, grain N yield, post-anthesis N uptake, and GPD (Fig. 4; Supplementary Fig. S2). As expected, the influence of the phenology parameters on anthesis date was independent of N supply; however, their effect on the other model outputs were highly dependent on N supply. In all site–N combinations, all the parameters associated with phenology had an effect of the same sign on anthesis date and crop DM at maturity, but of opposite sign on anthesis date and GY. In other words, early flowering crops had higher GY associated with higher DM accumulation after anthesis and DM harvest index. Three parameters associated with leaf layer expansion (AreaPL, NLL) and light interception (Kl) had some indirect effects on anthesis date due to non-linearity or interactions between parameters (Supplementary Fig. S2). This result could be explained by their effect on canopy temperature.

At CF and RR under HN, the green area index (GAI) at anthesis was essentially influenced in order of importance by the number (NLL) and the potential size (AreaPL and aSheath) of the leaves produced after floral initiation, and the potential ratio of the flag leaf to penultimate leaf size (RatioFLPL). Under these growing conditions, the potential phyllochronic duration between the end of expansion and the beginning of senescence of the leaves produced after floral initiation had some non-linear and/or interaction effects on GAI (Supplementary Fig. S2 at *JXB* online). Under dry conditions and non-limiting N supply NLL, P, AreaPL, MinL, and the FTSW threshold for the response of leaf expansion to water deficit (LowerFSTWexp) were the most influential parameters (in order of importance) for GAI at anthesis (Fig. 4; Supplementary Fig. S2). For most of the model outputs and parameters, the sensitivity indices were significantly higher under HN than under LN. The only significant exceptions were GAI at anthesis and total crop DM at maturity which were more influenced by the parameter related to N allocation under LN than under HN. Under LN, canopy expansion was primarily determined by N supply; therefore, it was most influenced by the N requirement of growing leaf tissues (CritSLN). It was also significantly influenced by RUE and Kl.

At all site–N combinations, RUE had the highest influence on GY. Under HN, at CF and RR the potential duration of grain filling (Dgf) was the second most influential parameter. In contrast, at AV, Dgf had no significant overall effect on GY. At the three sites under LN, the phyllochron was the second most influential parameter with respect to GY. At CF and RR, the potential number of culm leaves (NLL) had a significant overall effect on GY under both N treatments, while the potential size of the culm leaves (AreaPL) and Kl had significant overall effects on GY only under HN. NLL and GY were negatively related under all site–N combinations. At RR under LN, the relative rate of accumulation of grain structural DM during the endosperm cell division period (Kcd) and the duration of the endosperm cell division period (Dcd) were significantly associated with GY uncertainty. The parameters determining the response of RUE to the ratio of diffuse to direct light (SlopeFR), leaf N (TauSLN), temperature (Tmax and Topt), and water deficit (LowerFTSWrue and UpperFTSWrue) had much less effect on GY than the parameters mentioned above. At AV, the response of leaf senescence to water deficit (LowerFTSWsen and MaxDSF) and the capacity of the crop to extract soil water (MaxRWU) had a high overall effect on GY, especially under HN. None of the parameters associated with DM or N partitioning had a significant effect on GY.

As regards GY, at all site–N combinations, RUE was the parameter influencing GPC the most. The effect of the other parameters on GPC depended on both the site and the N supply (Fig. 3). The phyllochron was the second most important parameter for GPC at AV (under both N), while at the two other sites it was the potential duration of grain filling (Dgf) under HN and the number of culm leaves (NLL) under LN. At all site–N combinations, Kl and Dgf were among the five most influential parameters. Under HN at the three sites, the N storage capacity of the leaves (MaxSLN) and NLL had the strongest overall positive effect on GPD. This was obtained despite a negative effect of these two parameters on post-flowering N uptake. Under LN, RUE had a strong negative effect on GPD. Kl also had significant overall negative effects on GPD at the three sites. All the parameters with a significant influence on GY or GPC had opposite effects on these two variables. However, the phyllochron, at the three sites and both N treatments, the parameters related to the response of leaf senescence to water deficit (UpperFTSWsen and MaxDSF), and the capacity of the crop to extract soil water (MaxRWU), at AV and to a lesser extent at CF, had an effect of the same sign on both GY and GPD.

### Quantitative sensitivity analysis of the influential parameters

The Morris design is an efficient screening method to identify influential model parameters. However, it does not allow quantification of the interactions between the model parameters, and the Morris sensitivity indexes are only qualitative. Therefore, based on the Morris analysis, 41 influential parameters were identified (Table 4) and their individual first-order (*S*_i_) and total (*S*_Ti_, first-order plus interactions of any order with all the other parameters) effects were calculated using the E-FAST method (Saltelli *et al.*, 1999).

**Table 4.** Parameters of the wheat simulation model *SiriusQuality*2 selected through the Morris screening analysis for the E-FAST analysis

Submodel	Total no. of parameters	Selected parameters	
		No. of parameters	Name
Phenology	15	9	MinL, Lincr, Ldecr, P, Pincr, SLDL, PFLLAnth, VAI, VBEE
Leaf Layer Expansion	11	6	AreaPL, PlagLL, PsenLL, RatioFLPL, aSheath, NLL
Light Interception and Use Efficiency	7	5	Kl, TauSLN, SlopeFR, RUE, Topt
Grain	6	3	Dcd, Dgf, Kcd
DM Allocation	7	1	FracBEAR
N Allocation	15	9	CritSLN, MaxSLN, StrucLeafN, MaxStemN, StrucStemN, AlphaSSN, AlphaNNI, BetaKn, BetaSSN
Root Growth and N Uptake	5	3	MaxRWU, MaxNuptake, BetaRWU
Soil Drought	9	5	MaxDSF, UpperFTSWexp, UpperFTSWgs, UpperFTSWrue, UpperFTSWsen

**Fig. 5.**
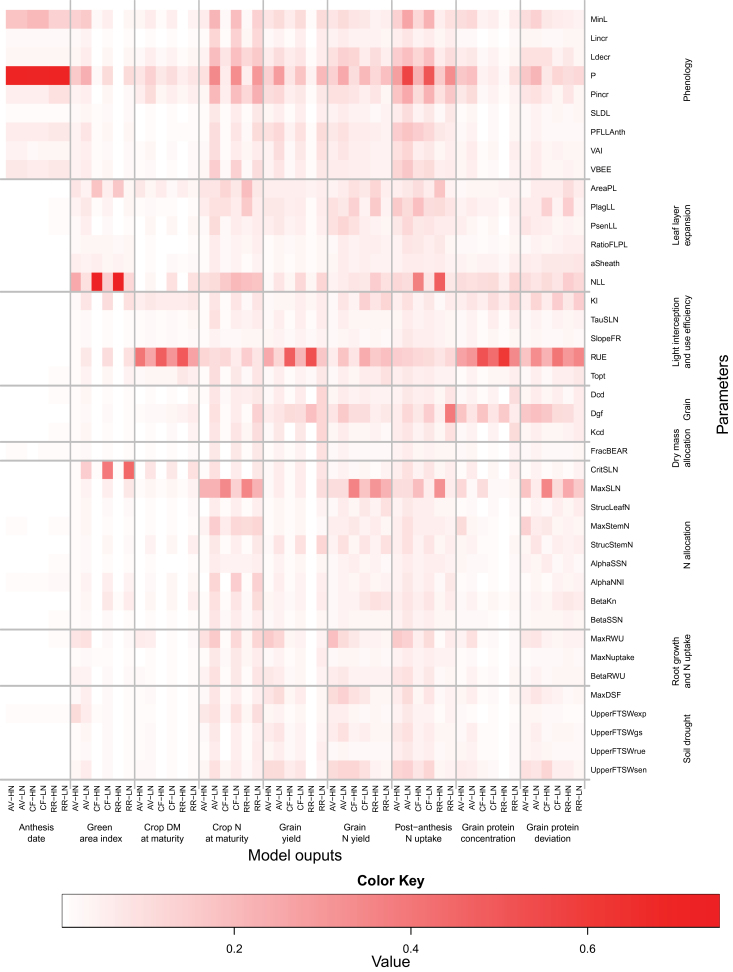
Heat map of the median values of the E-FAST total sensitivity index (*S*_Ti_) for the 41 input parameters selected from the Morris screening analysis of the wheat simulation model *SiriusQuality*2. *S*_Ti_ on anthesis date, green area index, crop DM at maturity, crop N at maturity, grain DM at maturity, grain N at maturity, post-anthesis N uptake, grain protein concentration, and grain protein deviation are shown. Simulations were performed at Avignon (AV), Clermont-Ferrand (CF), and Rothamsted (RR) under high (HN) and low N (LN) supply for 40 continuous years (1970–2009). The parameters were grouped according to the submodel to which they belong.

Although the number of parameter combinations generated in the E-FAST analysis was considerably higher than that generated in the Morris analysis, the range of variations in anthesis date, GY, and GPC was similar for the two methods (Supplementary Table S3 at *JXB* online). In general, parameter ranking for the two methods was in good agreement (Supplementary Fig. S3). The major difference was that in the Morris method the phenology parameters had greater effects on DM and N accumulation, GPC, and GPD under HN than under LN, while the opposite was observed with the E-FAST method (Figs 4, 5).

The analysis of the results of the E-FAST method was focused on the most influential parameters. The parameters accounting for 90% of the sum of *S*_Ti_ for GY, GPD, or GPC in at least 50% of the years and for at least one site and N treatment were selected. For each site–N treatment–model output combination, 6–17 parameters were selected, representing a total of 32 different parameters. Averaged across years these 32 parameters accounted for 48–87% of *S*_i_ for GY, GPC, and GPD, while the interactions between all of the parameters accounted for 8–56% of *S*_Ti_. For GY and GPC, the contribution of the interactions was 2-fold higher under LN (averaging 34%) than under HN (averaging 16%; Supplementary Fig. S4 at *JXB* online). At the different sites and for both N treatments, RUE and Dgf accounted for 22–71% of the sum of *S*_i_ with respect to GY, and GPC, except at AV where Dgf had no significant first-order effect on GY (Supplementary Fig. S4). At AV, maxRWU contributed 12% and 8% of the sum of *S*_i_ for GY under HN and LN, respectively. These results suggest that there are a few very influential parameters and a large majority of non-influential parameters. However, since the interactions between the parameters accounted for a large part of the total variance, the relative contribution of the different parameters to the variance of the model outputs cannot be quantified only from their first-order effect.

**Fig. 6.**
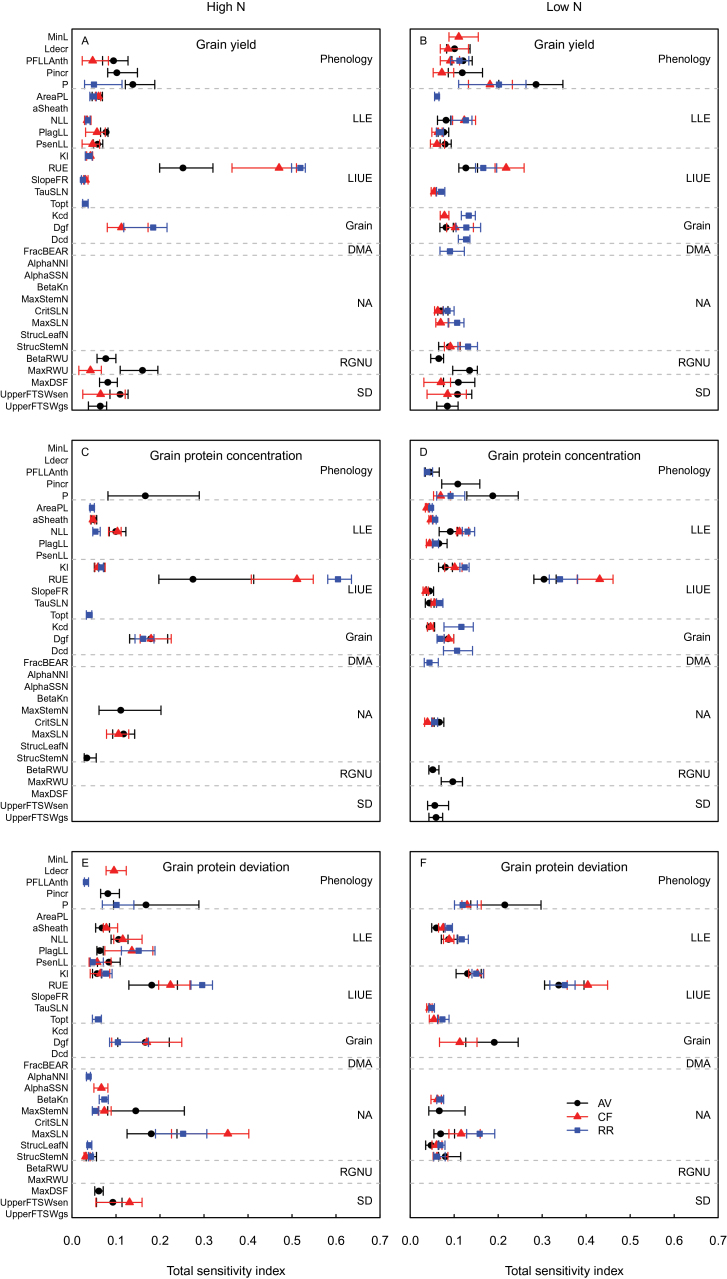
Plot of the median values of the E-FAST total sensitivity index (*S*_Ti_) for the 32 most influential parameters of the wheat simulation model *SiriusQuality*2 with respect to grain yield (A, B), grain protein concentration (C, D), and grain protein deviation (E, F) under high (A, C, E) and low (B, D, F) N supplies. Data include simulations at the three studied sites of Avignon (AV), Clermont-Ferrand (CF), and Rothamsted (RR) for 40 years (1970–2009). For each site, N treatment, and output variable, only the parameters contributing to 90% of the sum of the total sensitivity index in at least 50% of the years are plotted. Parameters are grouped according to the submodel to which they belong. The dashed horizontal lines delineate the different submodels: Phenology, Leaf Layer Expansion (LLE), Light Interception and Use Efficiency (LIUE), Grain, DM Allocation (DMA), N Allocation (NA), Root Growth and N Uptake (RGNU), and Soil Drought (SD). The error bars represent the 25% and 75% percentiles. (This figure is available in colour at *JXB* online.)

The *S*_Ti_ results showed that under HN the phenology parameters had much less influence on GY than estimated through the Morris screening analysis (Fig. 6A). At CF, GY was mainly influenced by the duration of the period between the appearance of the flag leaf ligule and anthesis (PFLLAnth), while at RR it was mainly influenced by the phyllochron. The ranking of the other parameters was similar to that observed in the screening analysis, and this quantitative analysis confirmed the primary role of RUE in determining GY under HN. As shown by the screening analysis, under LN, GY was determined by a large number of parameters (14–17 depending on the site) with equivalent effects (Fig. 6A). As for GY, the ranking of the parameters with respect to GPC and GPD was similar for the two methods (Fig. 6B, C). At AV under HN, the *S*_Ti_ value of the three most influential parameters (RUE, P, and MaxStemN) with respect to GPC and GPD showed a high dependency on the year, as indicated by the spread of the error bars.

## Discussion

Crop simulation models based on physiologically sound mechanisms have the potential to accommodate the uncertainty associated with gene and environment context dependencies (Campos *et al.*, 2004; Sinclair *et al.*, 2004; Cooper and Hammer, 2005; Hammer *et al.*, 2006). The analysis of system dynamics and properties through simulation experiments using such models provides a holistic approach to study soil–plant–atmosphere systems through the properties captured in the model. The in-depth simulation analysis carried out here contributes to the identification of traits with stable effects in a given population of environments needed to implement fully physiological trait-based breeding, for which phenotyping solutions are being developed (Lopes *et al.*, 2014; Pask *et al.*, 2014).

The results of the E-FAST analysis are quantitative for the 41 selected parameters analysed. However, the results might be taken as qualitative when considering the 75 parameters of the model, since possible interactions between the selected parameters and the remaining parameters that were unchanged in the analysis are not accounted for. However, since the 34 parameters that were unchanged in the E-FAST analysis were selected based on a screening Morris analysis, it is possible to be confident that the E-FAST results can be taken as quantitative when extended to the original (75-parameter) model.

In good agreement with the pleiotropic nature of the genetic and physiological antagonisms between GY and GPC (Aguirrezábal *et al.*, 2014), all the parameters had antagonist effects on these two traits. The present results show that GPC can be increased by increasing the N storage capacity of the leaves and to a lesser extent that of the stem. In most cases, and in good agreement with reported phenotypic correlations (Bogard *et al.*, 2010), a higher GPD was associated with a higher post-anthesis N uptake.

Overall, RUE and Dgf were the two parameters that influenced GY the most. Large genetic variability for RUE has been reported for wheat (García *et al.*, 2014), and genetic improvement in GY in the UK after 1983 has been positively associated with higher RUE (Shearman *et al.*, 2005). Genetic analyses have found genetic variations for the duration of grain filling of up to 40% for both bread (Charmet *et al.*, 2005) and durum (Akkaya *et al.*, 2006) wheat. Therefore, the present study supports earlier suggestions (Evans and Fischer, 1999; Reynolds *et al.*, 2012) that RUE and Dgf could contribute to improve GY in wheat. However, according to the results, Dgf had no significant effect on GY in Mediterranean environments with terminal water deficit. To a lesser extent, the effect of RUE on GY was also larger and more stable (less interannual variability) in wet than in dry environment.

Changing the number of culm leaves (NLL) independently of the final leaf number had a major influence on GY. With a constant phyllochron, increasing NLL could be an approach to increase the duration of the stem extension period, which has been suggested to increase ear dry mass at anthesis and grain number per square metre and thus yield potential (Slafer *et al.*, 2001; Foulkes *et al.*, 2011). However, in the present study, NLL was negatively associated with GY (i.e. increasing NLL decreased GY), and the influence of NLL on GY was higher under HN than under LN. This result is explained by the fact that even with 20g N m^–2^ of N fertilizer in HN, the higher early growth of simulated genotypes with higher NLL value depleted soil N, and the growth of the upper culm leaves was then limited by soil N supply. A higher number of culm leaves thus resulted in a reduction in the amount of light capture during the stem extension period which had a negative effect on ear growth and thus on grain number. The production of biomass during the grain-filling period was also reduced with higher NLL values.

Therefore, although increasing the number of culm leaves might be a good strategy to improve GY, it would require increasing the N supply and probably the number of fertilizer applications. A more precise monitoring of crop N needs would also be required so that leaf growth is not limited by N and applied N is not lost by leaching. This analysis of the effect of NLL is thus a good example where the complex response of the crop can hardly be understood through experimentation because of the complex interactions that occur. It is also an example where agronomic solutions should be developed along with genetic solutions in order to take full advantage of the genetic improvements.

Physiological constraints should also be considered when trying to manipulate plant development. Indeed changing the number of culm leaves independently of the final leaf number and therefore without affecting the anthesis date might be challenging. The leaf stage at the occurrence of the double ridge and terminal spikelet stage is tightly related to the final leaf number in wheat (Slafer *et al.*, 1994; Giunta *et al.*, 2001; Jamieson *et al.*, 2007). However, variations of about one leaf in this relationship have been observed between winter and spring near isogenic lines with the same final leaf number (Brooking and Jamieson, 2002). This result suggests that the range of variation tested in the present study (±0.9 leaf) could be achieved by genetics without changing the final leaf number and therefore the anthesis date.

Parameters related to phenology had a significant influence on almost all of the investigated model outputs and, as expected, their influence on GY was higher in dryer environments. In general, early flowering genotypes had higher GY because of a higher post-anthesis biomass production and higher harvest index. This trait has played a major role in the adaption of wheat to the Mediterranean climate (Richards, 1991). A recent analysis of genetic improvement in the CIMMYT spring wheat breeding programme has also shown a significant association of GY improvement with fewer days to anthesis (Lopes *et al.*, 2012). The importance of flowering time has been highlighted in many genetic analyses where co-locations of quantitative trait loci (QTLs) for flowering time and GY or GPC were found (e.g. Habash *et al.*, 2007; Laperche *et al.*, 2007). In particular, Bogard *et al.* (2011) showed that the phenotypic correlation between monocarpic leaf senescence and GY and GPD is largely explained by anthesis dates, as major QTLs for these traits co-locate with major flowering time QTLs. Consequently, the benefits of using populations with a narrow range of phenology to identify chromosomic regions associated with adaptive traits have been demonstrated (e.g. Olivares-Villegas *et al.*, 2007).

The present results show that phyllochron could be a valuable trait to improve GY. Among the phenology parameters, phyllochron appeared as the most influential. Phyllochron is a key determinant of early vigour, especially in growing conditions where yield is mainly determined by pre-anthesis biomass production (Rebetzke and Richards, 1999), which can in turn affect tillering through the carbohydrate supply/demand balance (Bos and Neuteboom, 1998; Rebolledo *et al.*, 2012). In the analysis reported here, under LN, phyllochron was the most important parameter determining GY at AV, and the second most important at the other two sites. The importance of phyllochron under LN was mainly due to its strong interactions with other parameters. Interestingly, phyllochron was the only parameter that had an effect of the same sign on both GY and GPD at the three sites. Significant genetic variations exist for phyllochron in wheat (He *et al.*, 2012), and specific QTLs were found in durum wheat (Sanna *et al.*, 2014), rice (Miyamoto *et al.*, 2004; Morita *et al.*, 2005), and barley (Borràs-Gelonch *et al.*, 2010).

If physiological traits are to be used in crop breeding programmes, there is a need to develop protocols for their efficient phenotyping. For phyllochron, Jamieson and Munro (1999) have proposed a simplified field-based protocol that could be implemented in a breeding programme. In contrast, determining the number of extended internodes and therefore the number of culm leaves might be more difficult to quantify with the necessary precision (Weightman *et al.*, 1997), and it might require determination of the size of the leaves associated with each phytomer under near optimal growth conditions (Fournier *et al.*, 2005), which in overwintering conditions would require at least two plant samplings. Regarding RUE, high-throughput protocols in the greenhouse are currently being developed and will provide a relevant alternative to field protocols that use punctual measurements of canopy light interception (Moreau *et al.*, 2012; García *et al.*, 2014). Finally, for Dgf, visual assessment of physiological maturity might be a valuable alternative to ear sampling (Hanft and Wych, 1982; Talbert *et al.*, 2001).

In the Morris analysis, μ* and σ were closely correlated. Therefore, a parameter that is important in the model is usually also involved in non-linear or interaction effects. In other words, the ‘overall’ importance of a model parameter is primarily determined by the non-linear response of the model or by its interactions with other model parameters. The interactions were quantified in the E-FAST analysis: 8–56% of the variance in GY, GPC, and GPD were accounted for by the interactions between the parameters. Therefore, the effect of a parameter cannot be accurately quantified based on its first-order effect. The effect of the parameters on GY was more balanced under LN than under HN. The higher trait×trait interactions found in the present study under LN compared with HN may explain, at least in part, the lower genetic progress in GY under LN than under HN (Le Gouis, 2011). This result has important implications for physiological breeding as the introgression of traits whose value depends on interactions with other traits in an improved genotype will be more difficult than in the case of traits with lower interactions. This also calls for the definition of ideotypes that combine several traits (Semenov and Stratonovitch, 2013; Martre *et al.*, 2014a).

The interactions between parameters found in this study depend on the hypothesis on which the model is founded regarding the interactions between physiological processes and forcing (environmental) factors for particular processes. Recent studies have shown that multimodel ensembles give more accurate predictions of GY (Asseng *et al.*, 2013; Bassu *et al.*, 2014) and of processes leading to yield than any individual model; that is, ensemble estimators have less compensation of errors than individual models and therefore causal relationships are more likely (Martre *et al.*, 2014b). The difficulty in the use of multimodel ensembles to identify putative traits that could be used for genetic improvement would be to achieve the correspondence between the parameters of individual models.

In summary, an in-depth simulation exercise was conducted using the wheat simulation model *SiriusQuality*2 to quantify the value of putative morpho-physiological traits for breeding and their environmental stability. To the best of the authors’ knowledge, it is the first study analysing the uncertainty of the whole set of parameters of a process-based crop simulation model. The results show that the overall effect of the parameters was dominated by their interactions with other parameters. Under non-limiting N supply, a few influential parameters with respect to GY were identified (e.g. RUE, Dgf, or P). However, under LN, >10 parameters showed similar effects on GY and GPC. All parameters had opposite effects on GY and GPC, but leaf and stem N storage capacity appeared as good candidate traits to shift the negative relationship between GY and GPC, confirming the results of an earlier analysis (Martre *et al.*, 2007).

## Supplementary data

Supplementary data are available at *JXB* online.

Figure S1. Long-term weather at the three sites considered in this study.

Figure S2. Heat map of the median values of the rescaled standard deviation of the distribution of elementary effect from the Morris screening analysis.

Figure S3. Comparison of parameter rankings obtained for the Morris analysis and for the E-FAST analyses.

Figure S4. Radar plots of the median values of the E-FAST first-order sensitivity index for the 32 most influential parameters with respect to grain yield, grain protein concentration, and grain protein deviation.

Table S1. Ranges of differences in anthesis date and percentage changes in grain yield and grain protein concentration explored in the Morris screening analysis.

Table S2. Statistics of the linear regression between simulated grain protein concentration and grain yield for the 760 parameters combinations of the Morris screening analysis.

Table S3. Ranges of differences in anthesis date and percentage changes in grain yield and grain protein concentration explored in the E-FAST analysis.

Supplementary Data

## Abbreviations:

AV: Avignon
CF: Clermont-Ferrand
DM: dry mass
E-FAST: extended Fourier amplitude sensitivity test
FTSW: fraction of transpirable soil water
GPC: grain protein concentration
GPD: grain protein deviation
GY: grain yield
HN: high nitrogen
LN: low nitrogen
RUE: radiation use efficiency
RR: Rothamsted
SLN: specific leaf nitrogen.
