# Supplementary material for: In silico system analysis of physiological traits determining grain yield and protein concentration for wheat as influenced by climate and crop management
Source: J Exp Bot. 2015 Mar 24;66(12):3581–98. doi: 10.1093/jxb/erv049 (PMC4463803; doi:10.1093/jxb/erv049)
Supplement: Supplementary Data [file supp_66_12_3581__index.html]

 In silico system analysis of physiological traits determining grain yield and protein concentration for wheat as influenced by climate and crop management — In silico system analysis of physiological traits determining grain yield and protein concentration for wheat as influenced by climate and crop management — Supplementary Data 

# *In silico* system analysis of physiological traits determining grain yield and protein concentration for wheat as influenced by climate and crop management

## Supplementary Data

Data files

**Files in this Data Supplement:**

- Supplementary Data - Supplementary Data
- Supplementary Data - Supplementary Data
- Supplementary Data - Supplementary Data
