# Supplementary figures and images for: In silico system analysis of physiological traits determining grain yield and protein concentration for wheat as influenced by climate and crop management
Source: J Exp Bot. 2015 Mar 24;66(12):3581–98. doi: 10.1093/jxb/erv049 (PMC4463803; doi:10.1093/jxb/erv049)

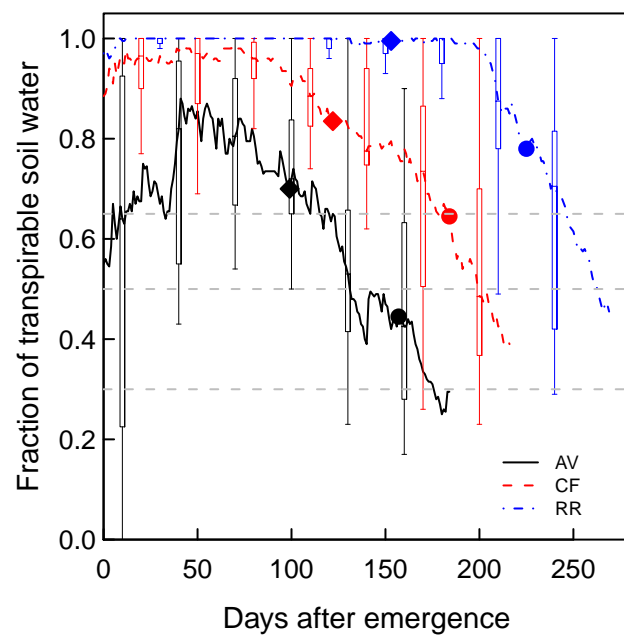

Supplement: Supplementary Data [file supp_erv049_jexbot141044_file002.pdf]

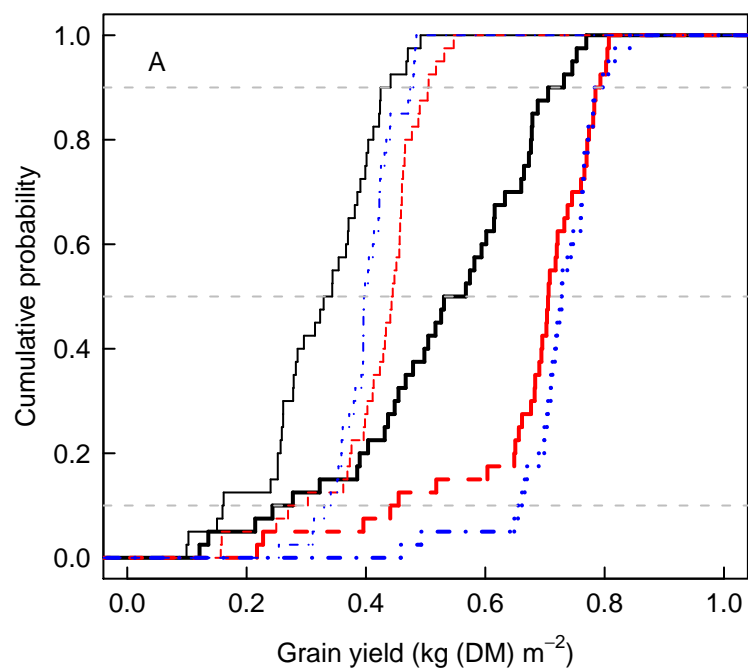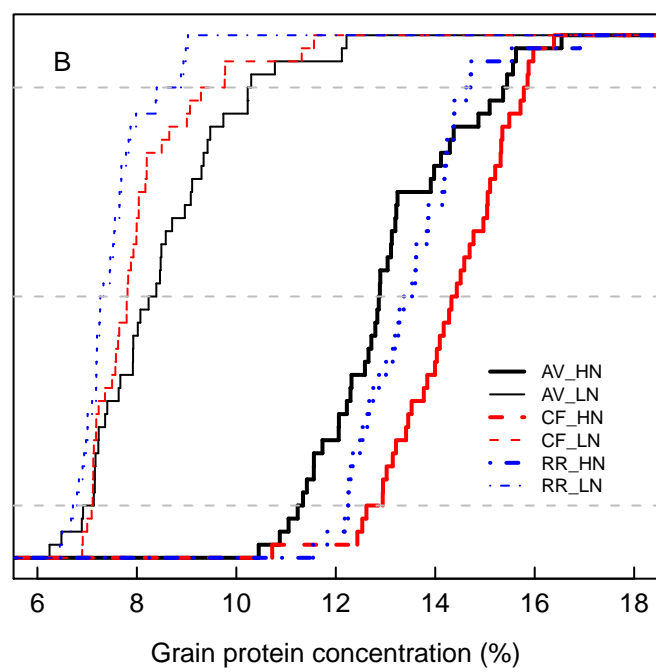

Supplement: Supplementary Data [file supp_erv049_jexbot141044_file003.pdf]
