# Supplementary material for: In silico system analysis of physiological traits determining grain yield and protein concentration for wheat as influenced by climate and crop management
Source: J Exp Bot. 2015 Mar 24;66(12):3581–98. doi: 10.1093/jxb/erv049 (PMC4463803; doi:10.1093/jxb/erv049)
Supplement: Supplementary Data [file supp_erv049_jexbot141044_file001.pdf]

Journal of Experimental Botany

Supplementary data

In silico system analysis of physiological traits determining grain yield and protein concentration for wheat as influenced by climate and crop management

Pierre Martre, Jianqiang He, Jacques Le Gouis and Mikhail A. Semenov

Table S1. Ranges of differences in anthesis date and percent changes in grain yield and grain protein concentration for the 760 parameter combinations generated in the Morris screening analysis compared with the default parameter set. Median and the 10% and 90% percentiles are reported. Percentiles are in square brackets.

**Site**	**N treatment**	**Differences in anthesis date (days)**	**Changes (%)**	
			**Grain yield**	**Grain protein concentration**
AV	HN	4 [-12 – 15]	-17 [-45 – 16]	-3 [-19 – 22]
	LN	4 [-12 – 15]	-13 [-41 – 15]	7 [-12 – 36]
CF	HN	4 [-11 – 17]	-11 [-29 – 14]	0 [-20 – 28]
	LN	4 [-10 – 17]	-8 [-30 – 9]	6 [-16 – 29]
RR	HN	4 [-14 – 16]	-8 [-25 – 16]	8 [-19 – 35]
	LN	4 [-13 – 16]	-8 [-26 – 10]	7 [-13 – 30]

Table S2. Statistics of the linear regression between simulated grain protein concentration and grain yield for the 760 parameter combinations of the Morris screening analysis. The median value of the slope and r^2 and the number of regressions above the indicated *P*-value for 40 continuous years (1970-2009) are reported. Values in square brackets are the 10% and 90% percentiles of the slope and r^2.

Site	N treatment	Slope (% protein g^{-1} (DM) m^2)	r^2	Number of significant regressions		
				$P < 0.001$	$P < 0.01$	$P < 0.05$
AV	HN	-1.29 [-11.27 – 5.26]	0.05 [0.00 – 0.38]	29	30	30
	LN	-15.55 [-19.33 – -10.91]	0.38 [0.28 – 0.65]	39	39	39
CF	HN	-13.64 [-19.92 – 5.08]	0.33 [0.02 – 0.63]	37	38	38
	LN	-13.60 [-17.56 – - 9.98]	0.49 [0.25 – 0.65]	40	40	40
RR	HN	-19.07 [-22.16 –-11.61]	0.75 [0.28 – 0.81]	38	39	39
	LN	-15.46 [-20.09– -12.82]	0.54 [0.42 – 0.61]	40	40	40

Table S3. Ranges of differences in anthesis date and percent changes in grain yield and grain protein concentration for the 29,889 parameter combinations generated in the E-FAST quantitative analysis compared with the default parameter set. Median and the 10% and 90% percentiles are reported. Percentiles are in square brackets.

**Site**	**N treatment**	**Differences in anthesis date (days)**	**Changes (%)**	
			**Grain yield**	**Grain protein concentration**
AV	HN	0 [-13 – 13]	-11 [-42 – 25]	-2 [-20 – 22]
	LN	0 [-13 – 13]	-11 [-37 – 16]	6 [-15 – 34]
CF	HN	1 [-12 – 14]	-8 [-30 – 16]	0 [-20 – 29]
	LN	1 [-12 – 15]	-10 [-26 – 11]	6 [-15 – 34]
RR	HN	0 [-14 – 15]	-5 [-26 – 18]	5 [-19 – 36]
	LN	0 [-14 – 15]	-8 [-25 – 12]	8 [-12 – 33]

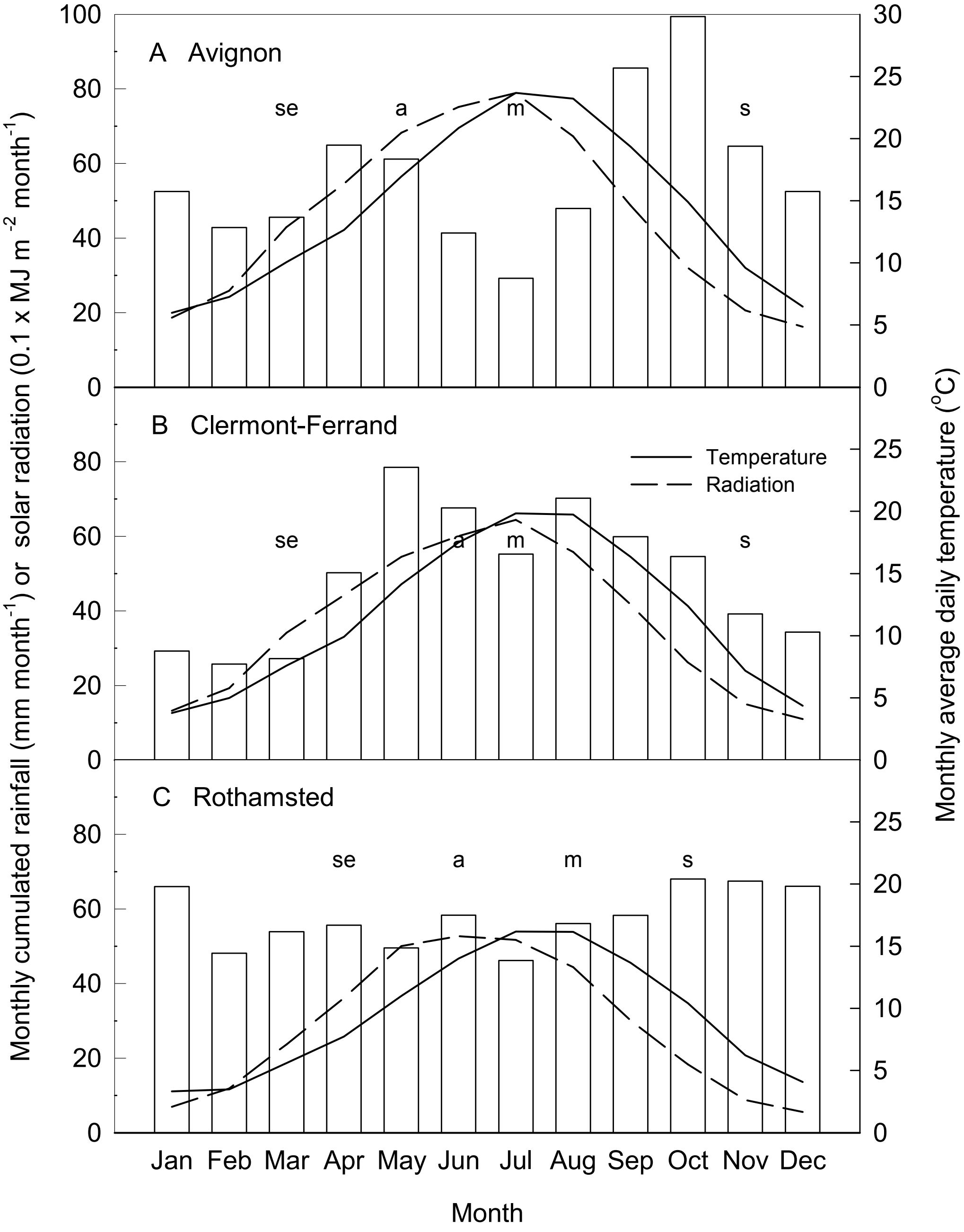

Fig. S1. Mean monthly total solar radiation (dashed lines), mean monthly daily temperature (solid lines), and mean monthly total rainfall (open bars) at Avignon (A), Clermont-Ferrand (B), and Rothamsted (C), for the 1970–2009 period. Letters indicate the median sowing (s), beginning of stem extension (se), anthesis (a), and crop maturity (m) dates for each site simulated by the wheat simulation model *SiriusQuality*2.

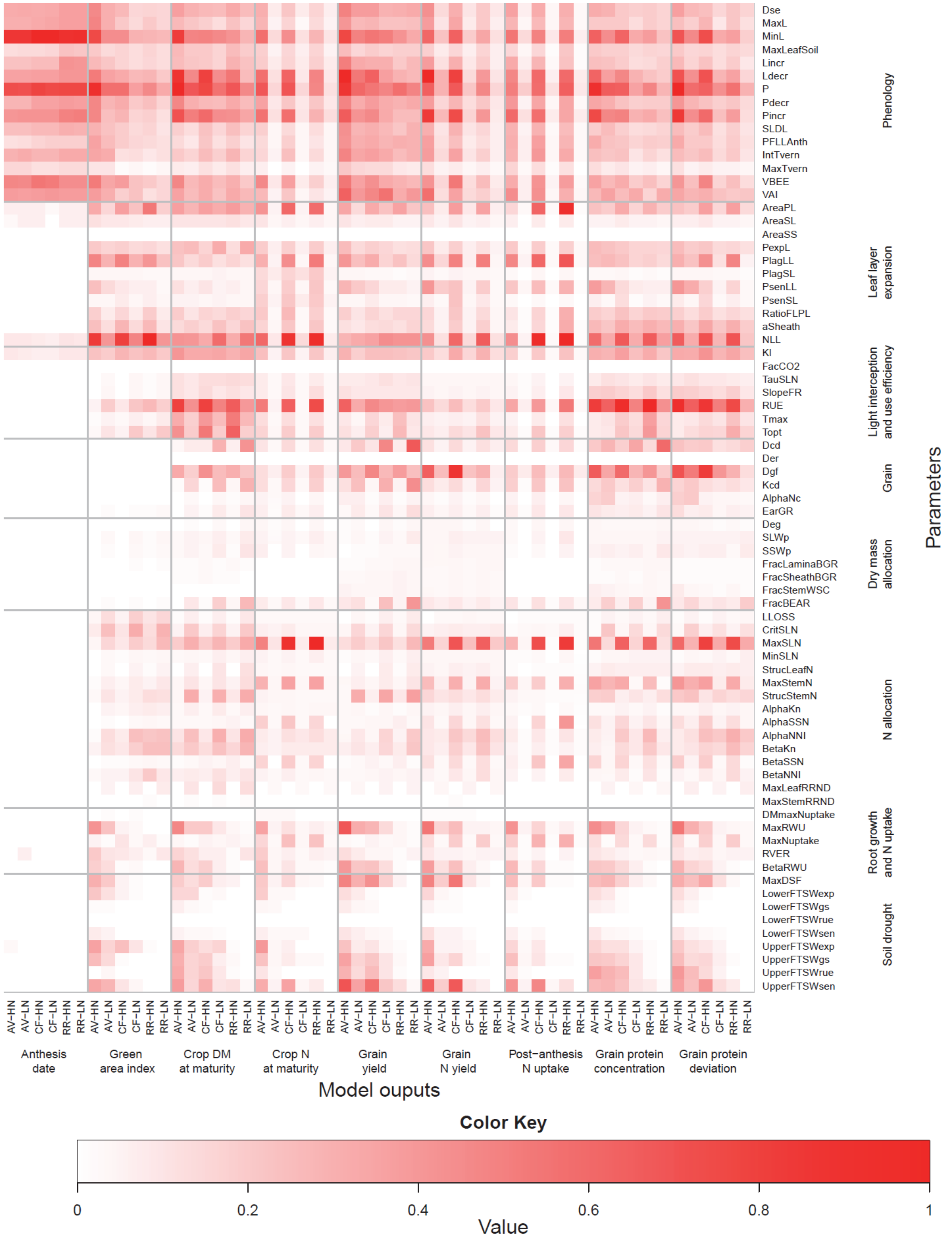

Fig. S2. Heat map of the median values of the rescaled standard deviation of the distribution of elementary effect (σ) from the Morris screening analysis of the 75 input parameters of the wheat simulation model *SiriusQuality*2 on anthesis date, green area index, crop DM at maturity, crop N at maturity, grain DM at maturity, grain N at maturity, post-anthesis N uptake, grain protein concentration, and grain protein deviation. Simulations were performed at

Avignon (AV), Clermont-Ferrand (CF), and Rothamsted (RR) under high (HN) and low N (LN) supply for 40 continuous years (1970-2009). The median of σ was rescaled to [0, 1] across the sites and N treatments so for a given output they can be compared across the sites and N treatments. The parameters were grouped according to the sub-model to which they belong.

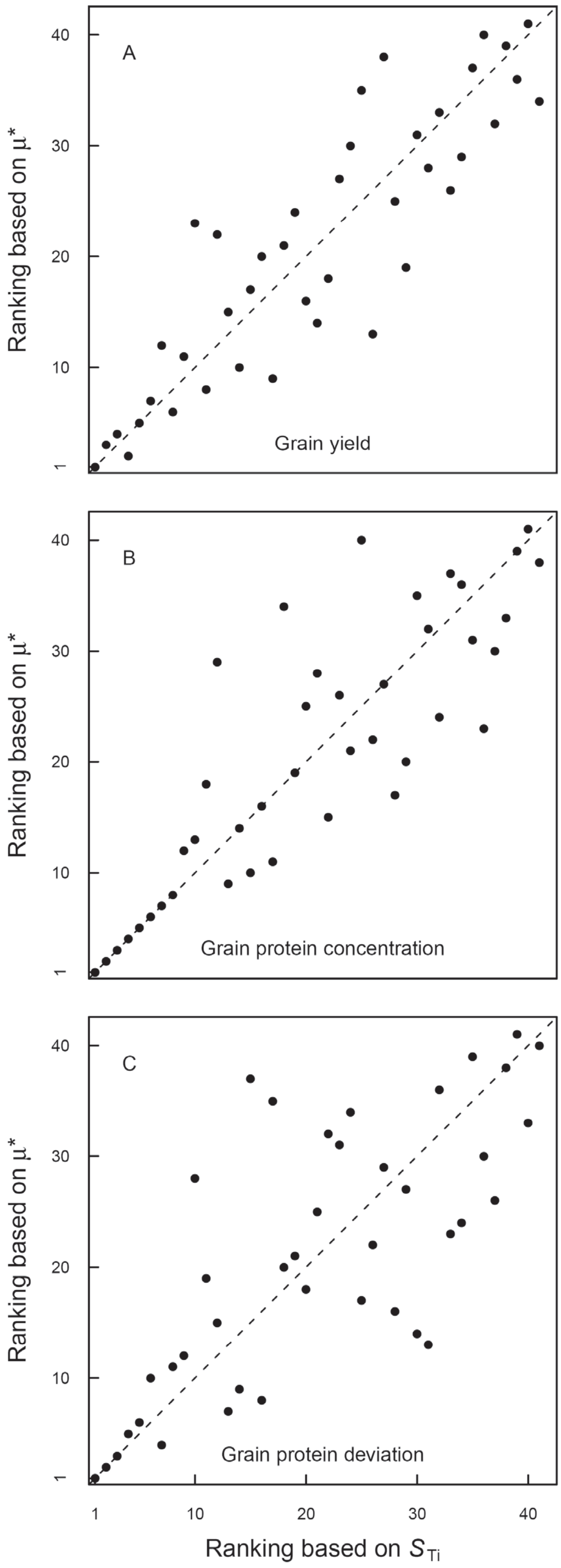

Fig. S3. Comparison of parameter ranking at Clermont-Ferrand under low N supply with respect to grain yield (A), grain protein concentration (B), and grain protein deviation (C) obtained for the Morris screening analysis (μ*) and for the E-FAST analysis (S_{Ti}). The most influential parameter is ranked 1.

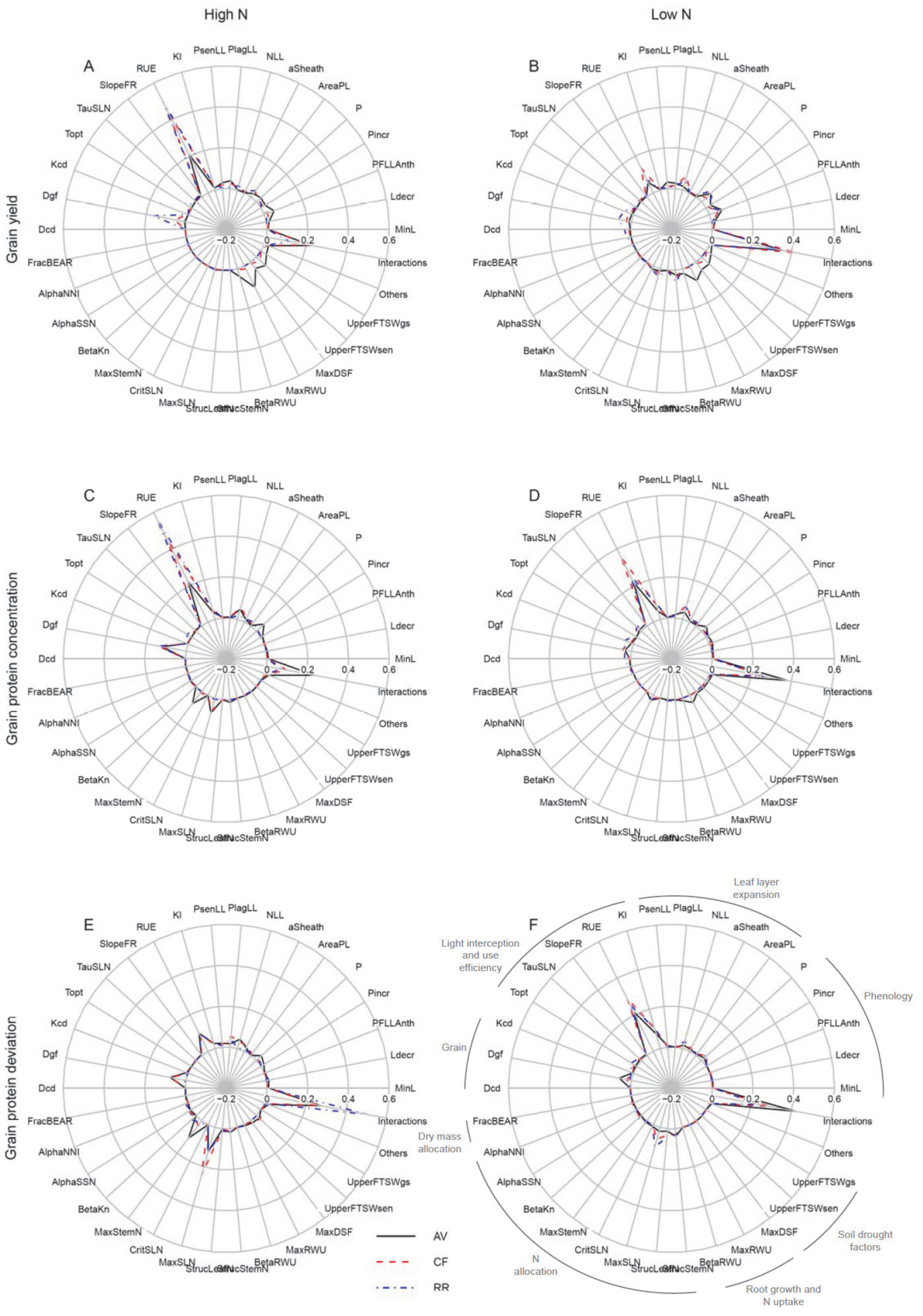

Fig. S4. (previous page) Radar plots of the median values of the E-FAST first-order sensitivity index (S_i) for the 32

most influential parameters with respect to grain yield (A, B), grain protein concentration (C, D), and grain protein deviation (E, F). Simulations were performed at Avignon (AV; solid lines), Clermont-Ferrand (CF; dashed lines), and Rothamsted (RR; dash-dotted lines) under high (A, C, E) and low (B, D, F) N supplies for 40 continuous years (1970-2009). For each site, N treatment, and output variable, only the parameters contributing to 90% of the sum of the total sensitivity index in at least 50% of the years are plotted. "Others" indicates the S_i contributed by the rest 9 parameters (out of the 41 used for the E-FAST analysis) not represented, while "Interactions" indicates the S_i contributed by interactions involving the 41 parameters. The parameters were grouped according to the sub-model to which they belong, as indicated in (F). The order of the parameters is conserved across the radar plots.
